# The complete chloroplast genome of *Epimedium tianmenshanense* (Berberidaceae)

**DOI:** 10.1080/23802359.2020.1720547

**Published:** 2020-02-06

**Authors:** Yue Zhang, Xiang Liu, Cheng Zhang, Baolin Guo, Chaoqun Xu, Guoan Shen, Lijuan Zhang

**Affiliations:** a Tianjin University of Traditional Chinese Medicine, Tianjin, PR China;; b Institute of Medicinal Plant Development, Chinese Academy of Medical Science, Peking Union Medical College, Beijing, China;; c Chongqing Academy of Chinese Materia Medica, Chongqing, PR China

**Keywords:** Chloroplast genome, phylogenetic analysis, *Epimedium tianmenshanense*

## Abstract

In this study, the complete chloroplast (cp) genome of *Epimedium tianmenshanense* was sequenced and assembled. The circular genome is 157,066 bp in size. The whole chloroplast genome of *E. tianmenshanense* contains 112 unique genes, of which 78 protein-coding genes, 30 tRNA genes, and four rRNA genes. The phylogenetic analysis based on 17 complete chloroplast genomes demonstrated a closer relationship between *E. tianmenshanense* and *E. lishihchenii*.

Epimedii Folium was used to treat hypertension, coronary heart disease, osteoporosis, etc. (Hu 2016; Ke et al. 2019; Zhang et al. 2019). In addition, Epimedii Folium also has anti-tumor, anti-aging, enhancing immunity, etc. (Lu et al. 2018; Luo et al. 2019). However, the classification and phylogenetic relationships among the *Epimedium* family have long been controversial because of the species diversity and their uneven geographical distribution of *Epimedium* species (Guo et al. 2007; Zhang et al. 2015; Yan et al. 2019). With the rapid development of technology, more and more researchers began to focus on the application of modern molecular techniques to characterize the genetic diversity of *Epimedium* family (Guo et al. 2018). Many study results have showed that the chloroplast genome sequences were essential data for plant phylogenetic and genetic population analyses (Parks et al. 2009). *Epimedium tianmenshanense,* a rare species, only occurs in the Tianmen Mountain of Hunan Province of China (Zhang et al. 2015). In the present study, we firstly reported the complete chloroplast genome of *E. tianmenshanense*, and characterized its structure features, which provides vital genetic and phylogenetic information for understanding the relationship of this species.

In this study, the samples of *E. tianmenshanense* were collected from the Zhangjiajie City of Hunan province in China (29°3′N, 110°28′E). A voucher specimen (Guo 17031) was deposited at the Herbarium of the Institute of Medicinal Plant, Chinese Academy of Medical Science, Beijing, China. The genomic DNA was extracted from the fresh leaves of *E. tianmenshanense* using the modified CTAB method (Doyle and Doyle 1987). DNA sequencing was performed on an Illumina Hiseq 2000 platform (Illumina Inc, San Diego, CA), and 150 bp paired-end reads were generated. The filtered reads were assembled into the complete chloroplast genome using the program GetOrganelle v1.5 (Jin et al. 2018) with *E. acuminatum* chloroplast genome (GenBank accession number: NC_029941) as a reference. The gene annotation of the chloroplast genome was conducted through the online program Geseq (Tillich et al. 2017) and CPGAVAS 2 (Shi et al. 2019), followed by manual correction if required. The finally annotated genomic sequence of *E. tianmenshanense* has been deposited in GenBank with the accession number MN882065.

The length of chloroplast genome of *E. tianmenshanense* was 157,066 bp. The large single copy (LSC) region, small single copy (SSC) region, and the two inverted repeat regions (IRs) of IRa and IRb, were 88,401, 17,021, and 25,822 bp in length, respectively. The GC content of complete chloroplast genome was 38.78%. The GC content of IR regions (43.18%) was higher than that in LSC (37.38%) and SSC regions (32.73%). A total of 129 functional genes were annotated. Among them, 112 genes, including 78 protein-coding genes, 30 tRNA genes and 4 rRNA genes, were unique in the chloroplast genome of *E. tianmenshanense*.

To get more knowledge about the phylogenetic status of *E. tianmenshanense,* we downloaded the complete chloroplast genomes of 17 plant species from the National Center for Biotechnoloyg Information (NCBI) to reconstruct a maximum-likelihood (ML) phylogenetic tree using the software of raxmlGUI1.5b (v8.2.10) (Silvestro and Michalak 2012). The results showed that *E. tianmenshanense* was closely related to *E. lishihchenii* (Figure 1). The complete chloroplast genome of *E. tianmenshanense* should be beneficial for further study on the taxonomy and systematics in the *Epimedium* family.

**Figure 1.**
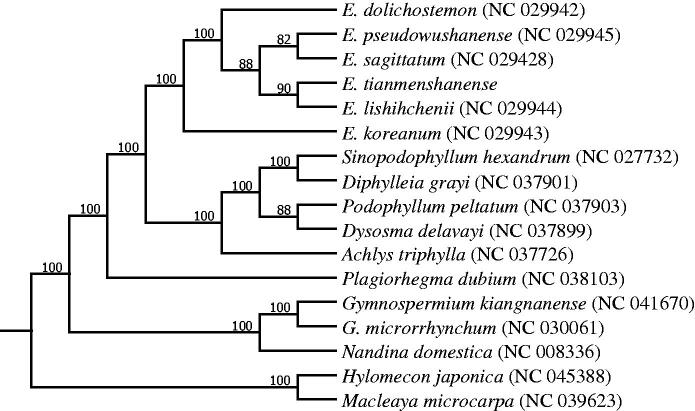
Maximum likelihood (ML) phylogenetic tree based on complete chloroplast genomes of 17 species, with *Hylomecon japonica* and *Macleaya microcarpa* as outgroup. Numbers above the lines represent ML bootstrap values.
